# Mind the pretzels and the pint: The PUB keeps you thirsty

**DOI:** 10.1093/plcell/koad194

**Published:** 2023-07-11

**Authors:** Andrew C Willoughby

**Affiliations:** Assistant Features Editor, The Plant Cell, American Society of Plant Biologists; Department of Biology, University of North Carolina at Chapel Hill, Chapel Hill, NC 27599, USA

When people enjoy salty snacks at a bar, their thirst can be quenched with an extra drink. The plant in the window, however, is unable to place an order. To cope with osmotic stress induced by drought conditions, plants rapidly increase their intracellular calcium and produce abscisic acid, a plant hormone involved in many stress responses. These signaling processes cause plants to close their stomata to prevent water loss and shape their root architecture to find more water—strategies needed for short- and long-term osmotic stress (Zhu 2016; Robbins and Dinneny 2018).

New work by **Wei Fan and colleagues (Fan et al. 2023)** deepens our understanding of the mechanisms underlying plant osmotic stress responses. Plant stress-induced signaling is often linked to increases in intracellular Ca^2+^ and the activation of calcium-dependent protein kinases (CPKs). CPK4 and CPK11 are activated under osmotic stress and promote abscisic acid signaling (Zhu et al. 2007). Fan et al. 2023 found that CPK4 protein levels are enhanced by osmotic stress but not by other environmental triggers such as temperature fluctuations. The authors show that CPK4 is degraded by the 26S proteasome and that osmotic stress boosts CPK4 activity by blocking this degradation (see Fig.).

**Figure.**
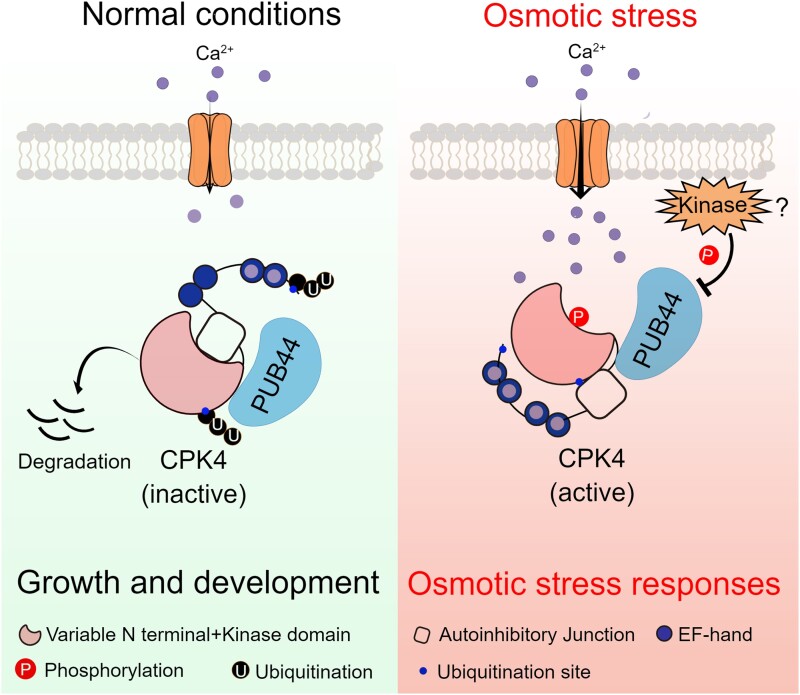
A model of how osmotic stress regulates CPK4 stability through PUB44 to promote signaling. Osmotic stress promotes Ca^2+^, activating CPK4. PUB44 degradation of CPK4 is relieved by CPK4 activation, leading to increased CPK4 stability and phosphorylation of downstream targets. Provided by W. Fan.

Identifying E3 ubiquitin ligases responsible for triggering proteasome degradation can be challenging, as interactions between the protein of interest and the ubiquitin ligase result in the degradation of the protein of interest. To overcome this limitation, the authors treated *CPK4-*overexpressing plants with a proteasome inhibitor and identified PUB44 as a CPK4-interacting partner by immunoprecipitation of CPK4 followed by mass spectrometry. PUB44 not only interacted with CPK4 but also was shown to ubiquitinate CPK4 in vitro and to negatively regulate CPK4 protein levels in vivo.

The authors found that regulation of CPK4 stability by PUB44 plays a role in osmotic stress responses. Plants that overexpressed *PUB44* had shorter roots compared with wild type when grown in osmotic stress conditions (mannitol media), showing that they are hypersensitive to the effects of osmotic stress on root elongation, whereas *pub44* mutants showed the opposite phenotype (longer roots under osmotic stress). The *pub44* phenotype was not apparent in *cpk4 cpk11 pub44* triple mutants. Therefore, PUB44 negatively affects osmotic stress–induced root growth inhibition, dependent on the presence of CPKs. However, these observations raise the question of how osmotic stress leads to an increase in CPK4 protein levels in wild-type plants. Interestingly, a kinase-dead variant of CPK4 was preferentially degraded by PUB44. The authors hypothesize that activated CPK4 is resistant to PUB44-mediated degradation due to a conformational change following CPK4 activation that shields the lysine residues targeted by PUB44 for ubiquitination. Thus, osmotic stress induces CPK4 activation and makes the protein resistant to PUB44-mediated degradation. Future structural biology work would shed light on this mechanism.

This work identifies the CPK4/PUB44 signaling module and how it allows plants to rapidly adjust CPK4 protein levels in response to osmotic stress. The authors demonstrate that PUB44 is capable of targeting CPK4 for degradation and that osmotic stress could shield CPK4 from interacting with PUB44 to promote downstream signaling.
